# Trends in mortality and disease burden from diabetes mellitus in Argentina between 1990 and 2022

**DOI:** 10.1590/1980-549720260039

**Published:** 2026-08-17

**Authors:** Camila Soledad Dominguez, Guillermo Raúl Macias

**Affiliations:** I Escola Nacional de Saúde Pública Sergio Arouca, Fundação Oswaldo Cruz – Rio de Janeiro (RJ), Brazil.; II Universidad Nacional de La Matanza – Buenos Aires, Argentina.

**Keywords:** Diabetes mellitus, Life expectancy, Epidemiology, Temporal distribution, Mortality, Argentina

## Abstract

**Objective:**

The study aimed to analyze the trends in mortality and disease burden due to Diabetes Mellitus in Argentina from 1990 to 2022.

**Methods:**

A longitudinal descriptive time-series study was conducted. Age-standardized mortality rates (ASMRs) and age-adjusted potential years of life lost (AAYPLL) were calculated. Trend analysis was performed using segmented regression (Joinpoint).

**Results:**

Between 1990 and 2022, a total of 270,394 deaths from diabetes mellitus were recorded in Argentina, of which 52.1% occurred among men and 48.8% among women. ASMRs and AAYPLL analyses consistently showed higher values among men than among women. The average annual percent change (AAPC) for both ASMRs and AAYPLL indicated a plateau among men and a significant decrease among women.

**Conclusion:**

The findings suggest a slight trend toward a decrease in the age at death among men, whereas the opposite pattern was observed among women. These findings have important implications for public health and policymaking. The high number of DM-related deaths among older age groups underscores the need for healthcare services tailored to the aging population. Furthermore, gender differences in mortality, with women tending to die at older ages than men, suggest that healthcare strategies should address gender-specific needs and risks.

## INTRODUCTION

Diabetes mellitus (DM) is one of the most prevalent noncommunicable diseases worldwide, and its patterns of morbidity and mortality have undergone substantial changes over the past century. According to the International Diabetes Federation, DM is among the fastest-growing global health emergencies of the 21^st^ century, affecting 537 million people worldwide in 2019 (projections reaching 783 million by 2045). In the same year, DM was associated with 6.7 million deaths. Approximately 3 out of every 4 adults living with DM reside in low- and middle-income countries^1^.

According to the World Health Organization (WHO), DM was the ninth leading cause of death worldwide in 2019^2^. In the same year, DM ranked as the sixth leading cause of death in the Region of the Americas^3^, with an estimated 409,000 deaths among adults aged 20 years old and older, accounting for 5.9% of all deaths. Between 1990 and 2019, the age-standardized rate of Disability-Adjusted Life Years increased by 27.4%, rising from 1,471.5 to 1,874.6 per 100,000 inhabitants. During the same period, mortality rates in the region increased by 19.0%, compared with a global average increase of 13.5%^4,5^.

Given its increasing prevalence and the concerns surrounding future projections, DM, as part of the broader group of noncommunicable diseases, has been incorporated into the 2030 Sustainable Development Goals (SDGs) agenda, which aims to reduce premature mortality from these diseases by one third.

Several studies have described the prevalence and mortality of DM in Latin American and Caribbean countries^6-10^, reporting high rates in recent years and, in some cases, projecting further increases driven by ongoing epidemiological, nutritional, and demographic changes.

In Argentina, data from the 2018 National Survey of Risk Factors showed that the self-reported prevalence of diabetes or elevated blood glucose increased from 9.8% in 2013 to 12.7% in 2018^11^. With respect to gender differences, mortality rates from DM increased among both men and women between 1990 and 2001, followed by a declining trend; however, rates remained consistently higher among men. Age was identified as a key factor in this pattern, as crude mortality rates among women were lower than age-adjusted mortality rates^7^.

In recent years, Argentina has undergone substantial social, political, and economic transformations that may influence population health. In addition, population aging, driven by changes in fertility patterns, growth in the older adult population, and shifts in epidemiological profiles, underscores the need for an updated analysis of diabetes-related mortality.

This study provides updated and comparable evidence on trends in mortality and the burden of disease attributable to DM in Argentina by integrating age-standardized rates, change-point analyses, and disease burden measures. Unlike previous studies, which were limited to shorter time periods or focused exclusively on mortality, the present analysis enables the identification of recent temporal changes and gender-specific differences, thereby providing an empirical basis for monitoring the epidemiological transition and informing diabetes prevention and control policies in the country. Understanding trends in DM-related mortality has important implications for health policy planning, the organization of healthcare services, the monitoring of public health targets, and the prioritization of interventions. Therefore, the objective of this study was to analyze trends in mortality and the burden of disease attributable to DM in Argentina from 1990 to 2022 and to identify potential inflection points.

## METHODS

A descriptive, observational, longitudinal time-series study was conducted. All deaths among individuals residing in Argentina between 1990 and 2022 were included. Cases with unspecified gender and/or age were excluded. Mortality data were obtained from the Directorate of Statistics and Health Information of the Ministry of Health (*Dirección de Estadísticas e Información de Salud* – DEIS). Population data were derived from the National Population, Household, and Housing Censuses conducted in 1991, 2001, 2010, and 2022 by the National Institute of Statistics and Censuses (*Instituto Nacional de Estadística y Censos* – INDEC), as well as from the corresponding intercensal estimates. For the period 1990–1996, deaths coded as 250.0 to 250.9 according to the International Statistical Classification of Diseases and Related Health Problems, Ninth Revision (ICD-9) were included in the analysis^12^. From 1997 onward, deaths coded as E10 to E14 according to the International Statistical Classification of Diseases and Related Health Problems, Tenth Revision (ICD-10) were included^13^. ICD-9 codes were converted to ICD-10 codes using the correspondence tables published by Cirera Suárez et al.^14^, who performed dual coding (manual and standardized international coding) of more than 85,000 deaths. The correspondence between revisions was established using international statistical methodology, including comparisons of causes of death classified under both revisions and the calculation of comparability ratios and kappa coefficients. This approach allowed the level of agreement and the statistical impact of the classification change to be quantified^14^. Vital statistics in Argentina have demonstrated high-quality indicators regarding the certification of the underlying cause of death and the recording of gender and age^15^. Therefore, no correction and/or imputation procedures were applied.

Crude mortality rates for DM were calculated using the estimated population as of June 30 of each year, stratified by gender and age. Age-standardized mortality rates were calculated using the direct standardization method. The 2010 Argentine census population, grouped into five-year age intervals, was used as the standard population^16^. Ninety-five percent confidence intervals (95%CI) for the age-standardized mortality rates were estimated using the "exact" method based on the Byar approximation, assuming a Poisson distribution^17^.

To estimate the impact of premature mortality attributable to DM, the Age-Adjusted Potential Years of Life Lost (AAYPLL) indicator was used, following the methodology proposed by DEIS^18^. Although the Disability-Adjusted Life Years (DALYs) indicator is recommended for assessing the overall burden of disease, it could not be calculated in this study because complete information on disability was not available throughout the analyzed time series. Therefore, the burden of disease was approximated through an analysis of premature mortality.

The life expectancy (LE) value used to calculate AAYPLL was set at 80 years for both genders. This threshold was selected because it is consistent with the LE of the study population, which in Argentina was 79.48 years for both genders in 2020^19^. The use of this threshold facilitates comparisons with recent literature, given the continuous increase in LE observed in many countries and the need to capture years of life lost at older ages without excluding deaths occurring shortly before the maximum observed life expectancy^20,21^.

Temporal trend analyses were performed using segmented regression (joinpoint regression), with estimation of the annual percent change (APC) and the average annual percent change (AAPC). In addition, corresponding 95%CI were calculated, and statistical significance was assessed (alpha=0.05) under the null hypothesis of no change in slope (*i.e*., zero joinpoints)^22,23^. A trend was considered significantly increasing when the APC or AAPC estimate and the lower bound of its 95%CI were >0. Conversely, a trend was considered significantly decreasing when the estimate and the upper bound of its 95%CI were <0. Trends were classified as stable when the 95%CI included zero.

Microsoft Excel was used for data management and consolidation. Crude mortality rates, age-standardized mortality rates, and AAYPLL, along with their corresponding 95%CI, were calculated using EpiDat version 4.2^24^. The APC and AAPC were estimated using the Joinpoint Regression Program, version 4.9.1.0, developed by the Division of Cancer Control and Population Sciences of the United States National Cancer Institute^25^.

### Data availability statement:

The complete dataset supporting the findings of this study is available from the corresponding author upon reasonable request. The dataset is not publicly available because it includes an integrated database compiled by the authors.

## RESULTS

Between 1990 and 2022, a total of 270,394 deaths attributable to DM were registered in Argentina, of which 138,492 occurred among men and 131,902 among women. Sixteen deaths recorded as having "indeterminate gender" were excluded from the analysis. Overall, men accounted for 51.2% of deaths and women for 48.8% during the study period. At the beginning of the series, however, deaths were more frequent among women (53% ♀ *vs*. 47% ♂). Between 1994 and 2010, the distribution by gender remained relatively similar, with minor fluctuations. From 2010 onward, the proportion of deaths among men exceeded that among women, reaching a peak of 54% in 2018. By the end of the study period, men accounted for 52.9% of deaths and women for 47.1%. The mean age at death was 71.5 years overall, corresponding to 69.8 years among men and 73.3 years among women (statistically significant difference: Student's *t*-test, p=0.0000). Analysis by age group showed that mortality increased with advancing age. Men accounted for a higher proportion of deaths up to 74 years of age, whereas the distribution was similar between sexes in the 75–80-year age group. Among individuals older than 80 years, deaths were substantially more frequent among women (Graphic 1).

**Graphic 1:**
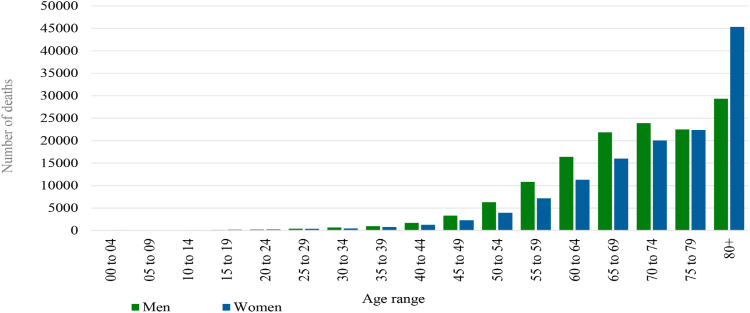
Deaths from Diabetes Mellitus by gender and age range. Argentina, 1990-2022.

Source: Prepared by the authors based on data from the Directorate of Health Statistics and Information.

At the national level, age-standardized mortality rates (ASMRs) remained consistently higher among men than among women throughout the study period (Graphic 2).

**Graphic 2:**
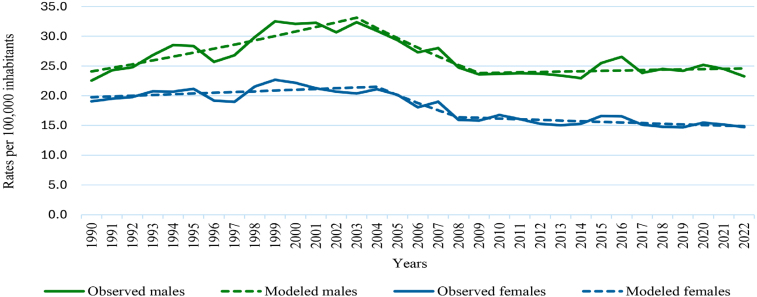
Trend in age-adjusted mortality rates for Diabetes Mellitus. Argentina, 1990-2022.

Source: Prepared by the authors based on data from the Directorate of Health Statistics and Information and the National Institute of Statistics and Census.

As shown in Graphic 2 and Table 1, ASMRs among women exhibited two inflection points. The first segment, spanning 1990 to 2004, showed an increasing trend. This was followed by a declining trend between 2004 and 2008, after which the rates remained stable. Among men, ASMRs increased until 2003, followed by a declining trend through 2009 and a subsequent period of stability.

**Table 1:** Trend analysis of age-adjusted mortality rates due to Diabetes Mellitus by gender. Argentina, 1990-2022.

Gender	Segment	Start Year	End Year	APC	95%CI	p-value
Males	1	1990	2003	2.48*	(1.76; 3.46)	0.000
	2	2003	2009	-5.34*	(-10.21; -3.22)	0.001
	3	2009	2022	0.25	(-0.44; 1.35)	0.424
Females	1	1990	2004	0.61*	(0.01; 1.58)	0.048
	2	2004	2008	-6.57*	(-9.85; -2.94)	0.006
	3	2008	2022	-0.69	(-1.33; 0.68)	0.179

* Statistically significant.

APC: Annual Percentage Change; CI: confidence interval.

Source: Prepared by the authors based on data from the Directorate of Health Statistics and Information and the National Institute of Statistics and Censuses.

Analysis of the AAPC in ASMRs attributable to DM revealed distinct patterns by gender. Among men, a slight upward trend was observed, with an AAPC of 0.06% (CI -0.15; 0.36), although this change was not statistically significant (p=0.4951). In contrast, women exhibited a significant declining trend, with an AAPC of -0.88% (CI -1.10; -0.59; p≤0.000001).

Analysis of AAYPLL also revealed consistently higher values among men than among women (Graphic 3). Among men, AAYPLL exhibited an initial upward trend through 2002, followed by a decline until 2009 and a subsequent period of increase. In contrast, AAYPLL among women remained stable until 2004, decreased significantly through 2008, and subsequently stabilized (Table 2, Graphic 3).

**Table 2:** Trend analysis of age-adjusted potential years of life lost due to Diabetes Mellitus mortality by gender. Argentina, 1990-2022.

Gender	Segment	Start Year	End Year	APC (%)	95%CI	p-value
Males	1	1990	2002	2.95*	(2.20; 4.00)	<0.001
	2	2002	2009	-4.57*	(-8.60; -2.90)	<0.001
	3	2009	2022	0.77*	(0.10; 1.70)	0.022
Females	1	1990	2004	0.49	(-0.30; 1.80)	0.112
	2	2004	2008	-5.04*	(-8.30; -1.00)	0.043
	3	2008	2022	-0.13	(-1.10; 2.70)	0.929

* Statistically significant.

APC: Annual Percentage Change; CI: confidence interval.

Source: Prepared by the authors based on data from the Directorate of Health Statistics and Information and the National Institute of Statistics and Censuses.

**Graphic 3:**
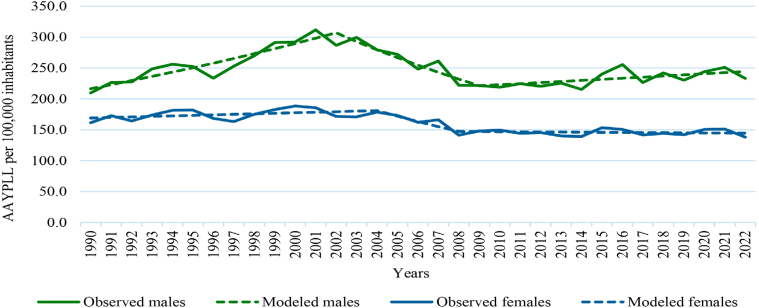
Trend in age-adjusted potential years of life lost due to Diabetes Mellitus mortality. Argentina, 1990-2022.

Source: Prepared by the authors based on data from the Directorate of Health Statistics and Information and the National Institute of Statistics and Censuses.

In contrast, the AAPC showed a significant increasing trend among men, with an AAPC of 0.38% (CI 0.20; 0.70; p=0.002). Among women, a significant decreasing trend was observed, with an AAPC of -0.49% (CI -0.80; -0.10; p=0.023). Both trends were statistically significant.

## DISCUSION

This study examined trends in mortality and the burden of disease attributable to DM in Argentina over a 33-year period, enabling the characterization of temporal patterns and the assessment of their sustained impact on population health. The findings indicate that ASMRs among men remained stable over time, whereas a slight decline was observed among women. With regard to AAYPLL, an upward trend was identified among men, while a downward trend was observed among women. These sex-specific differences in temporal trends are consistent with those reported in the National Diabetes Burden Profiles published by the Pan American Health Organization^26^.

When the present findings are considered alongside those of other studies conducted in the region, substantial heterogeneity in mortality and disease burden trends becomes evident. In Ecuador, for example, one study reported a significant increase in DM mortality among both men and women across most age groups and provinces during the period 2001–2016^10^. Similarly, a study conducted in the state of Paraná, Brazil, found that ASMRs doubled among both genders between 1984 and 2014, increasing from 14.37 to 29.27/100,000 inhabitants. Joinpoint analysis revealed a significant upward trend between 1984 and 2000, with an APC of 5.69% (4.24 to 7.16; p<0.01), followed by a stable trend from 2000 to 2014, with an APC of -0.08% (-1.09 to 0.94; p=0.87)^27^.

The results obtained for years of life lost due to diabetes AAYPLL during the study period indicate a slight trend toward a reduction in the age at death among men with DM (AAPC=0.38%). In contrast, the opposite pattern was observed among women, for whom an AAPC of -0.49% suggests that deaths occurred at older ages, reflecting a delay in DM-related mortality. When examining gender-specific differences reported in other studies conducted in the region, a study from Cuba is noteworthy, as it documented a marked decline in AAYPLL due to diabetes between 1987 and 2016, with higher AAYPLL rates among women^9^. Similarly, a study conducted in Chile for the period 1990–2000 reported a sustained and significant increase in APC among men, with an annual increase of 0.99% (95%CI 0.5; 1.5) throughout the study period. In contrast, women exhibited an annual decrease of 0.92% (95%CI -1.7; -0.1), trends similar to those observed in Argentina^8^. Overall, the studies conducted in the region do not demonstrate homogeneous patterns. However, the comparisons presented should be interpreted with caution because countries differ in their demographic transition processes and in the specific epidemiological profiles of their populations. Furthermore, the organization and accessibility of healthcare systems vary considerably across settings, potentially influencing the diagnosis, treatment, and severity of the disease.

Although it falls beyond the scope of the present study, it is important to note that several policies and strategies aimed at addressing DM were implemented during the study period and may have influenced the trends observed. At the national level, notable initiatives include Law No. 23.753 of 1989 on the diagnosis, treatment, and control of diabetes, regulated in 1998 through Decree No. 1.271; the establishment of the National Diabetes Program (*Programa Nacional de Diabetes* – PRONADIA) in 1999; National Law No. 25.501 enacted in 2001, which designated the control and prevention of cardiovascular diseases as a national health priority^28^; and the creation of the REMEDIAR Program in 2002, which guarantees coverage and free access to essential medicines. The latter program may have contributed to the decline in the trends of ASMRs and AAYPLL among men, and subsequently among women (Graphics 1 and 2). Nevertheless, more specific study designs would be required to evaluate the extent to which these interventions may have influenced changes in DM mortality.

Another relevant aspect for interpreting the findings concerns the risk factors associated with DM, many of which are shared with other chronic noncommunicable diseases, including tobacco use (and exposure to secondhand smoke), unhealthy diet, physical inactivity, and harmful alcohol consumption. According to the most recent National Survey of Risk Factors (*Encuesta Nacional de Factores de Riesgo* – ENFR) conducted in Argentina in 2018, the prevalence of self-reported excess weight (defined as the combined prevalence of overweight and obesity) increased from 40 to 61.6% across the four survey waves^1^. The prevalence of excess weight based on anthropometric measurements was even higher than the self-reported prevalence (66.1 *vs*. 61.6%). With respect to gender differences, the prevalence of self-reported excess weight was higher among men than among women (68.5 *vs*. 55.0%), which may have contributed to the gender-specific trends observed. Regarding blood glucose monitoring, the proportion of individuals reporting having undergone blood glucose measurement increased from 69.3% in the first survey to 79.1% in the most recent survey. In the latter survey, women reported a higher frequency of blood glucose measurement than men (83.8 *vs*. 73.9%)^11^.

Conversely, a decline in ASMRs was observed among both men and women beginning in 2020, coinciding with the onset of the COVID-19 pandemic. COVID-19 was the primary contributor to reductions in LE during the 2020–2022 period. During this time, gains in LE from other causes were observed, as many individuals died from conditions associated with the pandemic or from the indirect effects of public health measures, including social isolation, on other health outcomes^15^. Nevertheless, a comprehensive assessment of changes in DM mortality and their relationship with SARS-CoV-2 falls beyond the scope of the present study, particularly given the limited post-pandemic observation period available for analysis.

Although Argentina has extensive coverage and high availability of mortality data, one limitation of this study is the potential underreporting of DM as the underlying cause of death, as well as the underregistration of DM-related comorbidities^29^. Furthermore, the choice of threshold used to calculate AAYPLL may influence estimates of premature mortality and, consequently, affect their interpretation. In addition, the use of the same threshold for both genders may limit the ability to capture gender-specific differences in mortality patterns, highlighting the need for study designs aimed at identifying underlying determinants, particularly those related to prevention, healthcare, and access to and adherence to treatment. Similarly, the incorporation of socioeconomic factors and measures of healthcare access would contribute to a more comprehensive understanding of DM mortality. Finally, the national-level scope of the analysis limits the identification of subnational variations that may be relevant for informing public policies. Nevertheless, these limitations do not invalidate the findings, as the study provides a comprehensive overview of DM mortality and disease burden over the past three decades and identifies important avenues for future research.

Several questions and areas of debate remain open for advancing the understanding of DM trends and identifying factors that may contribute to either their reduction or worsening. Given that DM is a challenge shared by many countries, its study requires approaches that account for ongoing global transformations. Persistent inequalities in access to education, food quality, healthcare services, housing, and other social determinants contribute to disparities in quality of life and, consequently, to heterogeneous health profiles. In addition, population aging, together with the growth of the adult population, introduces gender-specific patterns and varying health conditions, placing DM in interaction with other health problems and increasing the complexity of its management^30^. Furthermore, the current context, characterized by substantial underfunding of preventive and healthcare policies, represents an important factor to be considered in future analyses of DM trends.

In this context, prioritizing studies that examine morbidity and mortality trends at subnational levels is essential, as such analyses may provide valuable evidence for the development of context-specific prevention and healthcare strategies.

The findings have important implications for public health and policymaking. First, the high number of deaths attributable to DM among older adults underscores the need for resilient healthcare systems, supported by strengthened chronic disease management and the adequate allocation of resources for long-term care.

Furthermore, the observed gender differences in mortality, with women tending to die at older ages than men, highlight the importance of incorporating a gender-sensitive approach into prevention and healthcare strategies, taking into account gender-specific risks, needs, and patterns of disease progression.

Fluctuations in mortality rates over time, characterized by periods of increase and decline, may help inform the evaluation of public health strategies, highlighting the importance of maintaining and replicating interventions associated with favorable trends.

Finally, the evidence generated may help inform resource allocation and the development of more effective interventions, reinforcing the importance of comprehensive, evidence-based approaches to improving population health outcomes.

## Footnotes

1 2005, 2009, 2013, and 2018 editions.
