# Supplementary material for: Trends in mortality and disease burden from diabetes mellitus in Argentina between 1990 and 2022
Source: Rev Bras Epidemiol. 2026 Aug 17;29:e260039. doi: 10.1590/1980-549720260039 (PMC13480621; doi:10.1590/1980-549720260039)
Supplement: Translation

# Translation: Tendencia de la mortalidad y carga de enfermedad por diabetes mellitus en Argentina entre 1990–2022

**DOI:** 10.1590/1980-549720260039.2

**Authors:** Camila Soledad Dominguez, Guillermo Raúl Macias

**Affiliations:** I Escola Nacional de Saúde Pública Sergio Arouca, Fundação Oswaldo Cruz – Rio de Janeiro (RJ), Brasil.; II Universidad Nacional de La Matanza – Buenos Aires, Argentina.

**Keywords:** Diabetes mellitus, Esperanza de vida, Epidemiología, Distribución temporal, Mortalidad, Argentina

## Abstract

**Objetivo:**

El trabajo propone analizar la tendencia de mortalidad y de la carga de enfermedad por Diabetes Mellitus en Argentina en el periodo 1990-2022.

**Métodos:**

Se realizó un estudio descriptivo longitudinal de series temporales. Se calcularon tasas de mortalidad ajustadas por edad (TAM) y Años de Vida Potencialmente Perdidos ajustados por edad (APVP-AE). El análisis de tendencia se desarrolló mediante regresión segmentada *(jointpoint)*.

**Resultados:**

Entre 1990 y 2022 fueron registrados en Argentina 270 394 óbitos por Diabetes Mellitus (DM), siendo el 52,1% varones y el 48,8% mujeres. Las TAM y el análisis de los APVP-AE muestra valores consistentemente más altos en varones que en mujeres. El porcentaje promedio de cambio anual (AAPC) de las TAM y de los APVP-AE muestran un estancamiento en varones y en mujeres una disminución significativa.

**Conclusión:**

Los resultados obtenidos indicarían una leve tendencia hacia la disminución en la edad de fallecimiento de los varones ocurriendo una situación inversa para las mujeres. Los hallazgos tienen importantes implicaciones para la salud pública y la formulación de políticas. El número de muertes por DM en los grupos etarios mayores subraya la necesidad de servicios de salud adecuados a la población adulta mayor. Además, las diferencias de sexo en la mortalidad, con mujeres que tienden a morir a una edad más avanzada que los hombres, sugieren que las estrategias de atención de salud deben considerar necesidades y riesgos específicos contemplando aspectos de género.

## INTRODUCCIÓN

La diabetes mellitus (DM) es una de las enfermedades no transmisibles más frecuentes, cuyos perfiles de mortalidad y morbilidad se han ido modificando a escala mundial en el último siglo. La Federación Internacional de Diabetes sostiene que es una de las emergencias sanitarias mundiales de más rápido crecimiento del siglo XXI siendo 537 millones las personas con DM en el mundo en el año 2019 (se estiman 783 millones para 2045) con 6,7 millones de muertes. Aproximadamente 3 de cada 4 adultos con DM viven en países de bajos y medianos ingresos^1^.

Según la Organización Mundial de la Salud (OMS), en el año 2019 la mortalidad por DM ocupaba el noveno lugar en el mundo^2^. Para el mismo año en la Región de las Américas, fue la sexta causa principal de muerte^3^, estimándose que un total de 409 000 adultos de 20 años o más murieron por DM, lo que representó el 5,9% de todas las muertes. El cambio porcentual de los Años de Vida Ajustados por Discapacidad estandarizados por edad fue del 27,4% entre 1990 y 2019, pasando de 1471,5 a 1874, 6 por 100 000 habitantes. Además, en el mismo período, las tasas de mortalidad en la región aumentaron un 19,0%, en contraste con un aumento promedio mundial del 13,5%^4,5^.

Debido a su incremento y a la preocupación por escenarios futuros, la DM como parte de las enfermedades no transmisibles, ha sido incluida en la agenda de los Objetivos de Desarrollo Sostenible (ODS) 2030, con la meta de reducir en un tercio la mortalidad prematura por dichas enfermedades.

Diferentes estudios describen la prevalencia y mortalidad por DM en países de América Latina y el Caribe^6-10^, mostrando valores elevados en los últimos años, algunos incluso pronosticando su aumento debido a cambios epidemiológicos, nutricionales y demográficos.

En Argentina, según la Encuesta Nacional de Factores de Riesgo del año 2018, la prevalencia de diabetes o glucemia elevada por autorreporte aumentó del 9,8% en 2013 al 12,7% en 2018^11^. Respecto a los diferenciales por sexo, las tasas de mortalidad por DM en el período 1990 a 2001 aumentaron en hombres y mujeres, para luego descender, siendo consistentemente más altas en varones. En ese sentido, la edad resultó un factor clave que evidenció en mujeres tasas brutas de mortalidad menores respecto a las tasas de mortalidad ajustadas por edad^7^.

En los últimos años, el país ha sufrido relevantes transformaciones sociales, políticas y económicas que influyen en las condiciones de salud de la población. Asimismo, la profundización del envejecimiento poblacional, relacionado con los cambios en los niveles de fecundidad, así como con el aumento de la población adulta mayor, y las mudanzas en los perfiles epidemiológicos, demandan un análisis reciente de la mortalidad por DM.

Este estudio aporta evidencia actualizada y comparable sobre la evolución de la mortalidad y la carga de enfermedad por DM en Argentina, integrando tasas estandarizadas por edad, análisis de puntos de cambio y medidas de carga de enfermedad. A diferencia de estudios previos limitados a periodos más cortos o centrados exclusivamente en mortalidad, permite identificar cambios temporales recientes y diferencias por sexo, proporcionando una base empírica para monitorear la transición epidemiológica y orientar políticas de prevención y control de diabetes en el país. El conocimiento de las tendencias de mortalidad por DM tiene importantes implicancias para la planificación de políticas, la organización de servicios de salud, el monitoreo de metas y la priorización de intervenciones. El objetivo de este trabajo es analizar la tendencia de mortalidad y de la carga de enfermedad atribuible a la Diabetes Mellitus en Argentina en el periodo 1990-2022, identificando puntos de inflexión.

## MÉTODOS

Se realizó un estudio descriptivo observacional longitudinal de series temporales. Se incluyeron en el estudio todos los óbitos de personas residentes en Argentina entre 1990–2022. Se excluyeron aquellos casos sin especificación de sexo y/o edad. Se utilizaron datos de mortalidad de la Dirección de Estadística e Información de Salud del Ministerio de Salud (DEIS), las poblaciones publicadas por el Instituto Nacional de Estadística y Censos (INDEC) de los Censos Nacionales de Población, Hogares y Viviendas de 1991, 2001, 2010 y 2022, y los cálculos intercensales respectivos. Para el período 1990–1996 se incluyeron en el análisis las muertes identificadas con los códigos de la 250.0 a 250.9 de la Clasificación Estadística Internacional de Enfermedades y Problemas relacionados con la Salud 9^a^ revisión (CIE-9)^12^, y a partir de 1997 los códigos E10 a E14 de la Clasificación Internacional de Enfermedades y Problemas Relacionados con la Salud 10^a^ revisión (CIE-10)^13^. Los códigos CIE-9 fueron convertidos a CIE-10 tomando como referencia las tablas publicadas por Cirera Suárez et al.^14^, quienes realizaron una doble codificación (manual y bajo normas internacionales) de más de 85.000 defunciones. La correspondencia fue establecida aplicando metodología estadística internacional (comparación de las causas de muerte bajo ambas revisiones calculando razones de comparabilidad e índice kappa), cuantificando la concordancia y el impacto estadístico del cambio de clasificaciones^14^. Las estadísticas vitales en Argentina presentan índices altos de calidad, tanto en la declaración de la causa básica de muerte como en la declaración de sexo y edad^15^, por lo que no se aplicaron métodos de corrección y/o atribución.

Se calcularon las tasas brutas de mortalidad por DM utilizando las poblaciones estimadas al 30 de junio de cada año según sexo y edad, y se ajustaron por método directo. La población estándar utilizada fue la población censal argentina de 2010 agrupada en grupos quinquenales de edad^16^. El cálculo de los intervalos de confianza del 95% (IC95%) de las tasas ajustadas de mortalidad se calculó de manera "exacta" mediante la aproximación de Byar, asumiendo distribución de Poisson^17^.

Para estimar el impacto de la mortalidad prematura por DM se utilizó el indicador Años Potenciales de Vida Perdidos Ajustados por Edad (APVP-AE), según la técnica propuesta por la DEIS^18^. Si bien se recomienda evaluar el impacto total de la enfermedad mediante el indicador Años de Vida Ajustados por Discapacidad (AVAD), en este caso no pudo ser calculado debido a la ausencia de información completa sobre discapacidad en la serie temporal analizada. Por ello, se intentó una aproximación al estudio de la carga de enfermedad mediante el análisis de la mortalidad prematura.

El valor de la esperanza de vida (EV) para ambos sexos a partir del cual se calcularon los APVP-AE, fue de 80 años. La elección de este umbral se realizó por situarse en consonancia con el rango de EV de la población estudiada, que en Argentina en el año 2020 fue de 79,48 años para ambos sexos^19^. La selección de este umbral permite una comparación más completa con la literatura reciente, dado el incremento continuo de la EV en muchos países y la necesidad de capturar la pérdida de vida aún en edades avanzadas sin excluir muertes que ocurren justo antes de la esperanza de vida máxima observada^20,21^.

El análisis de tendencia temporal se realizó mediante regresión segmentada (*joinpoint*), calculando el porcentaje de cambio anual (*Annual Percentage Change* –APC-) y el porcentaje promedio de cambio anual (*Average Annual Percent Change* –AAPC-). Además, se computaron los correspondientes IC95%, junto con su significación estadística (alfa=0,05), frente a la hipótesis nula de ausencia de cambio en la pendiente (cero *joinpoint*)^22,23^. Se considera como significativa una tendencia creciente si la estimación de APC o AAPC y su IC95% inferior son >0; como significativamente decreciente si la estimación y su IC95% superior son <0; y como estable si el IC95% contiene cero.

Para la consolidación de los datos se utilizó Microsoft Excel; las tasas brutas, las ajustadas por edad y los APVP-AE con sus intervalos de confianza del 95% fueron calculados con el programa EpiDat 4.2^24^. Para calcular el APC y el AAPC, se utilizó el programa *Joinpoint Regression*, Versión 4.9.1.0 de la División de Control de Cáncer y Ciencias de Población del Instituto Nacional del Cáncer de los Estados Unidos de América^25^.

### Declaración sobre la disponibilidad de datos:

El conjunto completo de datos que respalda los resultados de este estudio está disponible previa solicitud al autor correspondiente. El conjunto de datos no está disponible públicamente debido a la integración de la base de datos realizada por los autores.

## RESULTADOS

Entre 1990 y 2022, fueron registrados en Argentina 270 394 óbitos por DM, siendo 138 492 varones y 131 902 mujeres. Fueron exceptuados del análisis 16 casos identificados como "sexo indeterminado". El porcentaje de casos por sexo en el periodo analizado (51,2% ♂ y 48,8% ♀) comenzó siendo mayor en mujeres al inicio del periodo (53% ♀ vs. 47% ♂), entre 1994 y 2010 los valores se asemejan con algunas variaciones, y es a partir del año 2010, que el porcentaje de varones supera al de mujeres, observándose un pico del 54% en varones en el año 2018 y llegando a la actualidad al 52,9% y al 47,1% en mujeres. El promedio de edad al morir fue de 71,5 años, siendo de 69,8 en varones y de 73,3 en mujeres (diferencia significativa: Test t, valor de p=0,0000). Analizados por grupo etario, la mortalidad aumenta con la edad; los varones presentaron porcentajes mayores de defunciones hasta los 74 años, entre los 75 y los 80 años la frecuencia fue similar, y en la población mayor de 80 años la diferencia es marcadamente a favor de las mujeres (Gráfico 1).

**Gráfico 1:** Defunciones por Diabetes Mellitus según sexo y grupos de edad. Argentina, 1990-2022.

Fuente: Elaboración propia a partir de datos de la Dirección de Estadísticas e Información de Salud.

A nivel nacional, las tasas ajustadas de mortalidad (TAM) son consistentemente más altas en varones que en mujeres (Gráfico 2).

**Gráfico 2:** Tendencia de las tasas ajustadas de mortalidad por Diabetes Mellitus. Argentina, 1990-2022.

Fuente: Elaboración propia a partir de datos de la Dirección de Estadísticas e Información de Salud y del Instituto Nacional de Estadística y Censos.

Como se observa en el Gráfico 2 y Tabla 1, el porcentaje de cambio anual de las TAM en mujeres presenta dos puntos de inflexión. Un primer segmento corresponde al período 1990 a 2004 con una tendencia al aumento, un segundo momento que va hasta el año 2008 donde se observa un descenso, y un tercer período de estancamiento. Las TAM en varones presentan una tendencia inicial de aumento hasta el año 2003, seguida de una disminución hasta el 2009 y un tercer tramo de estancamiento.

**Tabla 1:** Análisis de la tendencia de las tasas ajustadas de mortalidad por Diabetes Mellitus según sexo. Argentina, 1990-2022.

Sexo	Segmento	Año inicio	Año fin	APC	IC95%	p-valor
Varones	1	1990	2003	2,48*	(1,76; 3,46)	0,000
	2	2003	2009	-5,34*	(-10,21; -3,22)	0,001
	3	2009	2022	0,25	(-0,44; 1,35)	0,424
Mujeres	1	1990	2004	0,61*	(0,01; 1,58)	0,048
	2	2004	2008	-6,57*	(-9,85; -2,94)	0,006
	3	2008	2022	-0,69	(-1,33; 0,68)	0,179

* Estadísticamente significativo.

APC: *Annual Percentage Change;* IC: intervalos de confianza.

Fuente: Elaboración propia a partir de datos de la Dirección de Estadísticas e Información de Salud y del Instituto Nacional de Estadística y Censos.

El análisis del porcentaje promedio de cambio anual (AAPC) de las TAM por DM revela diferencias significativas según sexo. Para varones se observa una ligera tendencia al aumento, siendo el AAPC de 0,06% (IC -0,15; 0,36), sin significancia estadística (valor de p=0,4951). Contrariamente, las mujeres presentan un AAPC negativo del -0,88% (IC -1,10; -0,59) siendo una disminución significativa (valor de p≤0,000001).

El análisis de los APVP-AE también muestra valores consistentemente más altos en varones que en mujeres (Gráfico 3). Puede observarse que los APVP-AE en varones presentan una tendencia inicial de aumento hasta el año 2002, seguida de una disminución hasta el año 2009 y un tercer tramo de aumento. En mujeres, se observa una tendencia estable hasta el año 2004, una disminución estadísticamente significativa hasta el año 2008 y una estabilización posterior (Tabla 2, Gráfico 3).

**Tabla 2:** Análisis de la tendencia de los Años de Vida Potencialmente Perdidos ajustados por edad por mortalidad por Diabetes Mellitus según sexo. Argentina, 1990-2022.

Sexo	Segmento	Año inicio	Año fin	APC (%)	IC95%	p-valor
Varones	1	1990	2002	2,95*	(2,20; 4,00)	<0,001
	2	2002	2009	-4,57*	(-8,60; -2,90)	<0,001
	3	2009	2022	0,77*	(0,10; 1,70)	0,022
Mujeres	1	1990	2004	0,49	(-0,30; 1,80)	0,112
	2	2004	2008	-5,04*	(-8,30; -1,00)	0,043
	3	2008	2022	-0,13	(-1,10; 2,70)	0,929

* Estadísticamente significativo.

APC: *Annual Percentage Change*; IC: intervalos de confianza.

Fuente: Elaboración propia a partir de datos de la Dirección de Estadísticas e Información de Salud y del Instituto Nacional de Estadística y Censos.

**Gráfico 3:** Tendencia de los Años de Vida Potencialmente Perdidos ajustados por edad por mortalidad por Diabetes Mellitus. Argentina, 1990-2022.

Fuente: Elaboración propia a partir de datos de la Dirección de Estadísticas e Información de Salud y del Instituto Nacional de Estadística y Censos.

Por otro lado, el AAPC para los varones muestra una tendencia al aumento del 0,38%, (IC 0,20; 0,70; valor de p=0,002) mientras que para las mujeres presenta una disminución del -0,49% (IC -0,80; -0,10; valor de p=0,023), ambos estadísticamente significativos.

## DISCUSIÓN

Este trabajo analiza la evolución de las tasas de mortalidad y la carga de enfermedad por DM en Argentina a lo largo de 33 años, permitiendo caracterizar patrones de cambio temporal y dimensionar su impacto sostenido sobre la morbimortalidad de la población. Según los resultados obtenidos, se observa que las TAM para el sexo masculino presentan un estancamiento a diferencia de las observadas en mujeres en donde existe un leve descenso. En cuanto a los APVP-AE, la tendencia en varones está en ascenso y en mujeres tiende a descender. Los diferenciales en las tendencias según sexo también pueden observarse en los Perfiles Nacionales de Carga de Enfermedad por Diabetes publicados por la Organización Panamericana de la Salud^26^.

Si analizamos los resultados presentados a luz de otros trabajos realizados en la región, se reconoce la existencia de heterogeneidad en las tendencias de mortalidad y carga de enfermedad. En el caso de Ecuador, por ejemplo, un estudio concluye que la misma se incrementó significativamente tanto para hombres como para mujeres en la mayor parte de los grupos etarios y provincias durante el periodo 2001–2016^10^. En otro estudio realizado en Brasil, en el Estado de Paraná, los resultados de las TAM dan cuenta de una duplicación de la mortalidad para ambos sexos en el período 1984 a 2014 de 14,37 para 29,27/100.000 habitantes y los resultados de *joinpoint* arrojaron crecimiento en el período de 1984 a 2000, con APC del 5,69% (4,24 a 7,16; p<0,01); manteniéndose estable entre el período de 2000 a 2014 con APC -0,08% (-1,09 a 0,94; p=0,87)^27^.

Los resultados obtenidos de los APVP-AE en el periodo estudiado indicarían una leve tendencia hacia la disminución en la edad de fallecimiento en varones con DM (AAPC=0,38%). En cambio, en mujeres se observa la situación inversa: el AAPC=-0,49% indicaría óbitos a edades mayores, retrasando la mortalidad por DM. Al observar los diferenciales por sexo en otros estudios realizados en la región, se destaca un trabajo realizado en Cuba donde se observan tendencias de APVP por DM durante el periodo 1987–2016 en notable descenso, con valores de las tasas de APVP más elevadas para el sexo femenino^9^. Por otro lado, un estudio realizado en Chile para el periodo 1990–2000 muestra un aumento sostenido y significativo de la APC en hombres con un 0,99% anual (IC95% 0,5; 1,5) durante todo el periodo y una disminución en mujeres del -0.92% anual (IC95% -1,7; -0,1), tendencias similares a las observadas en Argentina^8^. Por lo dicho, no se observan patrones homogéneos en los estudios realizados en la región. Sin embargo, es importante señalar que las comparaciones presentadas no deben interpretarse de manera lineal, debido a las diferencias entre los países en sus procesos de transición demográfica y los perfiles epidemiológicos propios de cada sociedad. Asimismo, la organización de los sistemas de salud y su grado de accesibilidad varían considerablemente, pudiendo influir en el diagnóstico, tratamiento y severidad del evento.

Si bien excede los alcances del presente artículo, es pertinente mencionar que durante el periodo analizado se desarrollaron diferentes políticas y estrategias para atender las problemáticas referidas a la DM que podrían haber tenido impacto en las tendencias analizadas. Se destacan, a nivel nacional: la Ley 23 753 del año 1980, relativa al diagnóstico, tratamiento y control de la enfermedad diabética, reglamentada en 1998 (Decreto 1271); el Programa Nacional de Diabetes (PRONADIA) en el año 1999, en 2001 la Ley Nacional 25 501 (que declara la prioridad sanitaria del control y la prevención de las enfermedades cardiovasculares en todo el territorio nacional)^28^, y la creación del programa REMEDIAR en el año 2002, que garantiza cobertura y acceso gratuito a medicamentos esenciales. Tal vez este último haya tenido influencia en el descenso de la tendencia de las TAM y los APVP-AE en varones, y un poco después en mujeres (Gráficos 1 y 2). Sin embargo, sería necesario realizar investigaciones con diseños más específicos para evaluar el impacto que dichas acciones pudieron tener en los cambios en la mortalidad por DM.

Otra cuestión relevante para la comprensión de los resultados radica en los factores de riesgo que se relacionan con la DM, muchos de los cuales se comparten con otras enfermedades crónicas no transmisibles, como son el consumo de tabaco (y la exposición pasiva), alimentación inadecuada, inactividad física y uso nocivo de alcohol. Según la última Encuesta Nacional de Factores de Riesgos (ENFR) realizada en Argentina en el año 2018, el exceso de peso por autorreporte (la suma del sobrepeso y la obesidad) aumentó a lo largo de las cuatro ediciones^1^ del 40 al 61,6%. La prevalencia de exceso de peso basándose en mediciones antropométricas fue aún mayor que la autorreportada (66,1 frente a 61,6%). Y con relación al sexo, la prevalencia de exceso de peso por autorreporte fue mayor en varones que en mujeres (68,5 frente a 55,0%), esto último podría influir en las diferencias por sexo observadas en las tendencias. Respecto a la población que refirió medición de glucemia, se observa un aumento del 69,3 al 79,1% entre la primera y última encuesta realizada, en donde las mujeres para el último período analizado reportaron mayor proporción de medición de la glucemia respecto de los varones (83,8 frente a 73,9%)^11^.

Por otro lado, se observa una disminución de las TAM en ambos sexos a partir del año 2020, momento en el que se desarrolla la pandemia por COVID-19. Dicha enfermedad ha sido la más influyente en la pérdida de EV durante el período 2020-2022, período en el que incluso se generan ganancias en la EV por otras causas, debido a que las personas afectadas fallecieron por enfermedades relacionadas con la pandemia o por los efectos que medidas de aislamiento tuvieron indirectamente sobre otros eventos de salud^15^. Sin embargo, supera las posibilidades de este trabajo realizar un análisis exhaustivo de los cambios de la mortalidad por DM y su relación con SARS-CoV-2, principalmente por los pocos años de análisis posterior a la misma.

A pesar de que el país presenta una elevada cobertura y disponibilidad de la información sobre mortalidad, una de las limitaciones del estudio se vincula al subregistro de la causa básica de muerte y las comorbilidades asociadas a la DM^29^. Asimismo, la elección del umbral para el cálculo de los APVP-AE condiciona las estimaciones de mortalidad prematura, pudiendo inducir interpretaciones erróneas. Además, el uso de un mismo umbral para ambos sexos impide captar diferencias en los patrones de mortalidad, por lo que se requieren diseños orientados a identificar determinantes específicos, particularmente en prevención y cuidado, así como en acceso y adherencia al tratamiento. En el mismo sentido, la incorporación de dimensiones socioeconómicas y de acceso a la salud, serían relevantes para comprender la mortalidad por DM. Por último, el análisis a nivel nacional limita la identificación de diferencias subnacionales, necesarias para orientar políticas públicas. Estas limitaciones no invalidan el estudio, que ofrece una visión global de la mortalidad y carga de enfermedad por DM en los últimos treinta años, y abre nuevas líneas de investigación.

Algunas preguntas y debates quedan abiertos para profundizar la comprensión de la tendencia de DM y cómo aportar a su disminución o agravamiento, considerando que es una problemática común a múltiples países y requiere enfoques que contemplen transformaciones globales. Por un lado, persisten desigualdades en el acceso a la educación, calidad de la alimentación, uso de los servicios de salud, vivienda y otras condiciones que determinan una calidad de vida diferencial y, en consecuencia, perfiles de salud heterogéneos. Por otro lado, el proceso de envejecimiento poblacional, asociado al aumento de la población adulta, introduce patrones diferenciales por sexo y condiciones de salud, y ubica a la DM en interacción con otras problemáticas de salud y mayores complejidades en su tratamiento^30^. Asimismo, el escenario actual, caracterizado por un marcado desfinanciamiento de las políticas de salud con enfoque preventivo y asistencial, constituye un aspecto central a considerarse en futuros análisis sobre el comportamiento de la DM.

En ese sentido, resulta prioritario profundizar en estudios que describan las tendencias de morbimortalidad en escalas territoriales menores, dado que podrían aportar evidencia útil para diseñar estrategias de prevención y atención situadas.

Los hallazgos presentan implicaciones relevantes para la salud pública y la formulación de políticas. En primer lugar, el elevado número de muertes por DM en los grupos de mayor edad subraya la necesidad de servicios de salud resilientes, mediante una mejor gestión de enfermedades crónicas y una asignación adecuada de recursos para los cuidados a largo plazo.

Además, las diferencias por sexo observadas en la mortalidad, con mujeres que tienden a morir a edades más avanzadas que los hombres, sugieren la necesidad de incorporar un enfoque de género en las estrategias de atención, considerando riesgos y necesidades específicas.

Las variaciones en las tasas de mortalidad, con periodos de aumento y descenso, pueden contribuir a interpretar los resultados de distintas estrategias, destacando la importancia de sostener y replicar aquellas que fueron exitosas

Finalmente, la evidencia generada puede orientar la asignación de recursos y el diseño de intervenciones más efectivas, reforzando la necesidad de enfoques integrales y basados en datos para mejorar los resultados de salud pública.

## Footnotes

1 Ediciones 2005, 2009, 2013 y 2018.
